# Introduction of H2C2‐type zinc‐binding residues into HIV‐2 Vpr increases its expression level

**DOI:** 10.1002/2211-5463.12358

**Published:** 2017-12-19

**Authors:** Ryoko Koga, Minami Yamamoto, Halil Ibrahim Ciftci, Masami Otsuka, Mikako Fujita

**Affiliations:** ^1^ Department of Bioorganic Medicinal Chemistry Faculty of Life Sciences Kumamoto University Japan; ^2^ Research Institute for Drug Discovery School of Pharmacy Kumamoto University Japan

**Keywords:** cysteine, HIV‐2, protein expression, Vpr, Vpx, zinc

## Abstract

Human immunodeficiency virus type 2 has two structurally similar proteins, Vpx and Vpr. Vpx degrades the host anti‐viral protein SAMHD1 and is expressed at high levels, while Vpr is responsible for cell cycle arrest and is expressed at much lower levels. We constructed a Vpr mutant with a high level of expression by replacing the amino acids HHCR/HHCH with a putative H2C2‐type zinc‐binding site that is carried by Vpx. Our finding suggests that during the evolution of Vpr and Vpx, zinc‐binding likely became a mechanism for regulating their expression levels.

**Abbreviations**
HIV: human immunodeficiency virus
PMA: phorbol 12‐myristate 13‐acetate
PPM: poly‐proline motif
SAMHD1: SAM and HD domain containing deoxynucleoside triphosphate triphosphohydrolase 1
SIV: simian immunodeficiency virus
VSV‐G: vesicular stomatitis virus G protein
WT: wild‐type

The amount of each protein in an organism is often strictly controlled and both insufficiently expressed and overexpressed proteins can cause abnormalities. This phenomenon is seen in microorganisms also. For example, the appropriate amount of Vif, one of the accessory proteins of HIV type 1 (HIV‐1), is maintained through ubiquitination followed by proteasome degradation 1, 2. When the proteasome degradation is too fast and the expression level of Vif is low, Vif does not work to confer viral infectivity 1, 3, 4. In contrast, if Vif is overexpressed, it inhibits Gag maturation 5.

Vpr and Vpx, are accessory proteins of HIV and simian immunodeficiency virus (SIV). In spite of sharing an ancestor and having a similar structure, the functions of these protein have diverged 6, 7, 8, 9 during the evolution of HIV/SIV through frequent cross‐species transmission 10. The expression levels of these proteins correspond to their different functions, and, as such, they are very different from each other. Interestingly, in HIV type 2 (HIV‐2), Vpx and Vpr have more than 20% homology 11 and are carried by the same gene, but they have different expression levels from one another in the context of the full genome: the amount of Vpx is much larger than that of Vpr 11, 12. This difference in expression levels relates to the different functions of these two proteins; for example, Vpx degrades the host anti‐viral protein SAM and HD domain containing deoxynucleoside triphosphate triphosphohydrolase 1 (SAMHD1), but HIV‐2 Vpr does not 9, 13, 14, and HIV‐2 Vpr arrests cells at the G_2_ phase, but Vpx does not 15, 16, 17, 18. However, the structural basis for the low expression level of HIV‐2 Vpr remains elusive.

Previously, we focused on the C‐terminal poly‐proline motif (PPM) that is carried by Vpx but not Vpr, and we constructed a chimeric HIV‐2 Vpr carrying the PPM of Vpx 19. However, this mutation did not increase the expression of Vpr. In the current study, we succeeded in generating a mutant that highly expresses HIV‐2 Vpr containing a putative zinc‐binding motif, like Vpx, showing that protein expression level regulation through the usage of zinc may have been important in the evolution of Vpr/Vpx.

## Materials and methods

### Plasmids

An expression vector for HIV‐2 Vpx (GH‐123 20/GL‐AN 21 strain in which an *Ava*I‐*Nco*I fragment of pGH‐123 was replaced by an *Ava*I‐*Nco*I fragment of HIV‐2_ROD_ and the *vpx/vpr* sequence originated from pGH‐123: GenBank accession no. M30895) with a FLAG tag at its N terminus (pEF‐Fvpx) and an expression vector for HIV‐2 Vpr (GH‐123/GL‐AN strain) with a FLAG tag at the same position (pEF‐Fvpr2) were previously constructed 19. An expression vector for HIV‐2 Gag (GH‐123/GL‐AN strain, the *gag* sequence originated from pGH‐123) with a FLAG tag at its N terminus (pEF‐Fgag2) was also previously constructed 22. Each mutation, 2KR, NWW, R83C, H38L, H76A or C81A, in the *vpr* sequence was introduced in the *Eco*RV–*Xba*I fragment of pEF‐Fvpr2 inserted into pBluescript SK(+) (Agilent, Santa Clara, CA, USA) by QuikChange site‐directed mutagenesis (Agilent), and the fragment was cloned into pEF‐Fvpr2 to yield each mutant. Expression vectors for Vpr of the different HIV‐2 strains, ROD10 23 and NMC842F‐1G 24, with a FLAG tag at their N terminus (pEF‐FRvpr2 and pEF‐FNvpr2) were constructed by replacement of pEF‐Fvpx with the *vpr* fragments carrying an *Eco*RV site at the 5′ terminus and an *Xba*I site at the 3′ terminus, amplified by PCR using pROD10 23 and pNMC842F‐1G 24, respectively. H83C mutation in the *vpr* sequences was introduced in the *Eco*RV–*Xba*I fragments of pEF‐FRvpr2 and pEF‐FNvpr2 inserted into pBluescript SK(+) by QuikChange site‐directed mutagenesis, and the fragments were cloned into pEF‐Fvpx to yield H83C mutants of pEF‐FRvpr2 and pEF‐FNvpr2, respectively. As infectious clones, pGL‐AN 21 and its *vpr*‐minus mutant pGL‐Ec 25 were used. To construct the pGL‐AN incorporating the H38L or R83C mutation, the mutation in the *vpr* region was introduced in the *Xba*I–*Nsi*I fragment of pGL‐AN inserted into pBluescript SK(+) (Agilent) by QuikChange site‐directed mutagenesis (Agilent). Each fragment was cloned into pGL‐AN to yield its mutant, pGL‐rH38L or pGL‐rR83C. To examine infectivity, an *env*‐deficient luciferase reporter GL‐AN clone (pGL‐Ns/luc) and its *vpx*‐minus mutant (pGL‐Ns/St/luc) 26 were used. The pGL‐Ns/luc incorporating the H38L or R83C mutation was constructed by inserting the *Pma*CI–*Pma*CI fragment of each pGL‐AN mutant into the two *Pma*CI sites of pGL‐Ns/luc. To generate vesicular stomatitis virus G protein (VSV‐G) pseudotyped virus, pCMV‐G 27 was used.

### Cell culture, differentiation and transfection

The human kidney cell line 293T 28 was maintained in Dulbecco's modified Eagle's medium supplemented with 10% heat‐inactivated fetal bovine serum, and the human monocytic cell line THP‐1 29 was cultured in RPMI‐1640 supplemented with 10% heat‐inactivated fetal bovine serum and 55 μm 2‐mercaptoethanol. THP‐1 cells were differentiated by stimulation with phorbol 12‐myristate 13‐acetate (PMA; Sigma‐Aldrich, St Louis, MO, USA) at 300 nm and incubation for 3 days. Transfection of 293T cells with various plasmids was performed using Lipofectamine 3000 (Life Technologies, Carlsbad, CA, USA), unless otherwise stated. Transfected cells were incubated for 2 days post‐transfection before use in experiments.

### Immunoblot analysis

Immunoblot analysis was conducted using cells lysed in PBS–Laemmli sample buffer (1 : 1), as described previously 1. As an antibody, anti‐FLAG M2 (Sigma‐Aldrich; 1 : 1000), anti‐β‐actin clone AC‐15 (Sigma‐Aldrich; 1 : 1000), HIV‐2 Vpx monoclonal antibody 6D2.6 (NIH AIDS Research and References Reagent Program) 30 (1 : 1000), or antiserum to SIV‐p27 (NIBSC Centralized Facility for AIDS reagent; 1 : 1000) was used. Immunoreactivity was detected by chemiluminescence using ImmunoStar Zeta (for detection of p27) or ImmunoStar LD (for detection of the other proteins; Wako Pure Chemical Industries, Osaka, Japan).

### *In vitro* transcription/translation and immunoprecipitation

For *in vitro* transcription/translation, the TNT T7 Quick Coupled Transcription/Translation system (Promega, Madison, WI, USA) using a rabbit reticulocyte lysate was used. To conduct immunoprecipitations, the reaction mixture was diluted (20×) in TNE buffer (10 mm Tris/HCl, pH 7.8, 0.15 m NaCl, 1 mm EDTA, 1% NP40, 10 μg·mL^−1^ aprotinin) and anti‐FLAG M2 Affinity Gel from mouse (Sigma‐Aldrich) was added. The suspension was incubated at 4 °C for 20 h. The gel was washed and analyzed by western blotting.

### ELISA, virion preparation, infection and luciferase assay

Virus amounts were determined by SIV p27 antigen ELISA kit (ZeptoMetrix, Buffalo, NY, USA). Virions were prepared for immunoblot analysis using Viro‐Adembeads (Ademtech, Pessac, France). Cells were infected with VSV‐G pseudo virus normalized to 600 ng of p27, in the presence of DEAE‐dextran (5 μg·mL^−1^; PK Chemicals, Køge, Denmark). The luciferase assay was performed using the Luciferase Assay System (Promega).

## Results

### Attempts to generate highly expressed Vpr mutant

**Figure 1:**
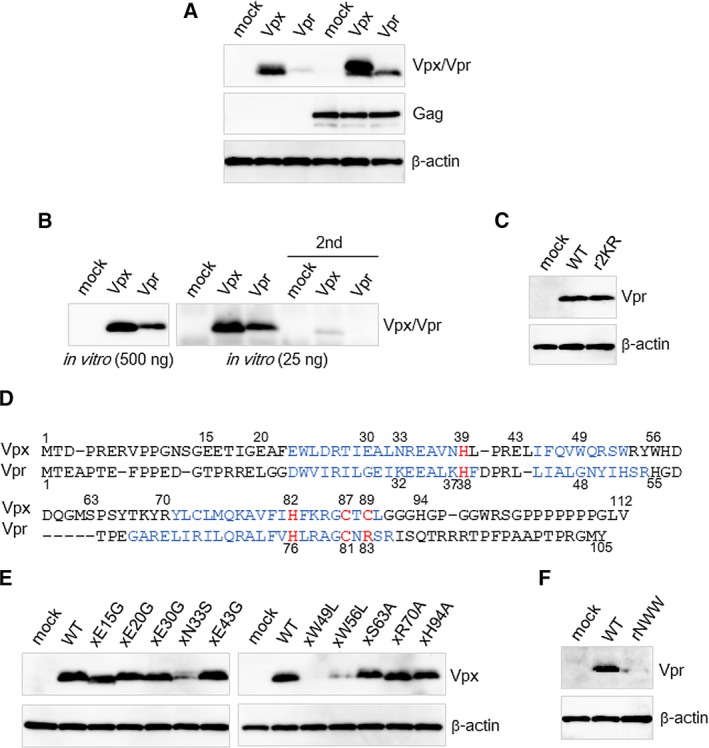
Expression of HIV‐2 Vpx/Vpr in WT and mutants. (A) Immunoblot analysis of proteins in 293T cells transfected with an expression vector for HIV‐2 Vpx with a FLAG tag at its N terminus (pEF‐Fvpx) or that for HIV‐2 Vpr with a FLAG tag at its N terminus (pEF‐Fvpr2) 19 (25 ng) along with the empty vector pEF1/*Myc*‐HisA or an expression vector for HIV‐2 Gag with a FLAG tag at its N terminus (pEF‐Fgag2) 22 (475 ng). (B) Immunoblot analysis of proteins obtained after *in vitro* transcription/translation using pEF‐Fvpx or pEF‐Fvpr2 (500 or 25 ng). When 25 ng of plasmid was used, immunoprecipitation using an anti‐FLAG gel was performed before the western immunoblotting. The supernatant of the immunoprecipitation was further incubated with the anti‐FLAG gel, followed by western immunoblotting (2nd). (C) Immunoblot analysis of proteins in 293T cells transfected with pEF‐Fvpr2 or its 2KR mutant (25 ng). (D) Sequence alignment of HIV‐2 (GH‐123/GL‐AN strain) Vpx and Vpr, previously performed in 11. Blue indicates those amino acids corresponding to predicted helices, and red shows amino acids of the H2C2 motif of Vpx and their corresponding amino acids in Vpr (all amino acids shown in red are on the helices). (E) Immunoblot analysis of proteins in 293T cells transfected with pEF‐Fvpx or its mutants (25 ng). (F) Immunoblot analysis of proteins in 293T cells transfected with pEF‐Fvpr2 or its NWW mutant (25 ng). In the transfections shown in (C, E, F), pEF1/*Myc*‐HisA (475 ng) was added in addition to pEF‐Fvpx/pEF‐Fvpr2 or its mutants (25 ng). In the blotting shown in (A–C, E, F), anti‐FLAG M2 was used to detect Vpx, Vpr and Gag, and anti‐β‐actin clone AC‐15 was used to detect β‐actin. The pEF1/*Myc*‐HisA was used as the mock sample.

Previous work using sole expression from a plasmid vector also found a lower amount of HIV‐2 Vpr protein than of Vpx protein 11, 19. Because Gag increases the protein amounts of Vpr 31 and Vpx 22, we initially performed a similar experiment with or without the expression of HIV‐2 Gag. An expression vector for HIV‐2 Vpx (pEF‐Fvpx) or that for HIV‐2 Vpr (pEF‐Fvpr2) 19 was transfected into 293T cells with or without the cotransfection of an expression vector for HIV‐2 Gag (pEF‐Fgag2) 22. The expression level of Vpx protein expressed by 293T cells transfected with 25 ng of pEF‐Fvpx by this method is thought to be similar to the expression level of Vpx by a full‐genomic infectious clone 22, and thus 25 ng of pEF‐Fvpx or pEF‐Fvpr2 and 475 ng of pEF‐Fgag2 or pEF1/*Myc*‐HisA were used for the transfections. The cells were lysed and then analyzed by western immunoblot analysis (Fig. 1A). Without Gag, the expression level of Vpx was much higher than that of Vpr, in agreement with previously reported results 11, 19. In the presence of Gag, the amounts of both Vpx and Vpr increased, but Vpx was still expressed more highly than Vpr.

The *in vitro* transcription/translation processes of Vpr and Vpx were then examined followed by a western immunoblot analysis. Expression levels were examined following transcription/translation with 500 or 25 ng of the vectors. When 25 ng of each vector was used, immunoprecipitation was performed before the western immunoblotting because the resulting expression levels were very low 22. The supernatant from the immunoprecipitation was further incubated with the anti‐FLAG gel to confirm that nearly all proteins were precipitated in the first immunoprecipitation experiment. As shown in Fig. 1B, the amount of protein expression is smaller for Vpr than it is for Vpx; however, this difference is less than the difference in expression level that was observed in cells (Fig. 1A). These results suggest the possibility that post‐translation degradation in a cell occurs more rapidly for Vpr than for Vpx, which is in agreement with the results of previous work 12.

We hypothesized that the observed degradation was caused by the proteasome after HIV‐2 Vpr was ubiquitinated. HIV‐2 Vpr (GH‐123/GL‐AN strain) has two lysines, both of which can be ubiquitinated, at its 32nd and 37th amino acids. Thus, we constructed an expression vector for a Vpr mutant carrying arginines instead of these lysines, which we named 2KR. The vector was transfected into 293T cells, and the expression level was compared with that of wild‐type (WT). However, no difference was observed between the Vpr expression level of WT and that of 2KR (Fig. 1C).

We next introduced into Vpr amino acids that were presumed to be associated with the normal expression of Vpx based on our previous mutational analysis 32. Among the 19 mutants used in that prior study, the two that contained mutations in the PPM region were excluded in light of earlier work indicating that the introduction of PPM to Vpr does not increase its expression 19. We first focused on the 14 mutants that were not zinc‐binding site mutants 22. Based on an alignment of Vpx and Vpr 11 (Fig. 1D), the amino acids in Vpr that correspond to these 14 amino acids in Vpx were checked, and the non‐homologous 10 amino acids of Vpx, E15, E20, E30, N33, E43, W49, W56, S63, R70 and H94, were selected. Since 25 ng of vector was recently shown to be a suitable amount for evaluating the expression of Vpx 22, the Vpx expression level of each mutant was examined in 293T cells transfected with 25 ng of pEF‐Fvpx mutants. As shown in Fig. 1E, N33S, W49L and W56L each had a lower expression level than that of WT, demonstrating that N33, W49, and W56 are important for the normal expression of Vpx. Each corresponding amino acid of Vpr, K32, G48 and H55, was then changed to N, W and W, respectively, constructing a Vpr NWW mutant carrying three point mutations. However, the Vpr expression level of this mutant was lower than that of WT (Fig. 1F).

### High expression of Vpr mutant carrying HHCC motif

**Figure 2:**
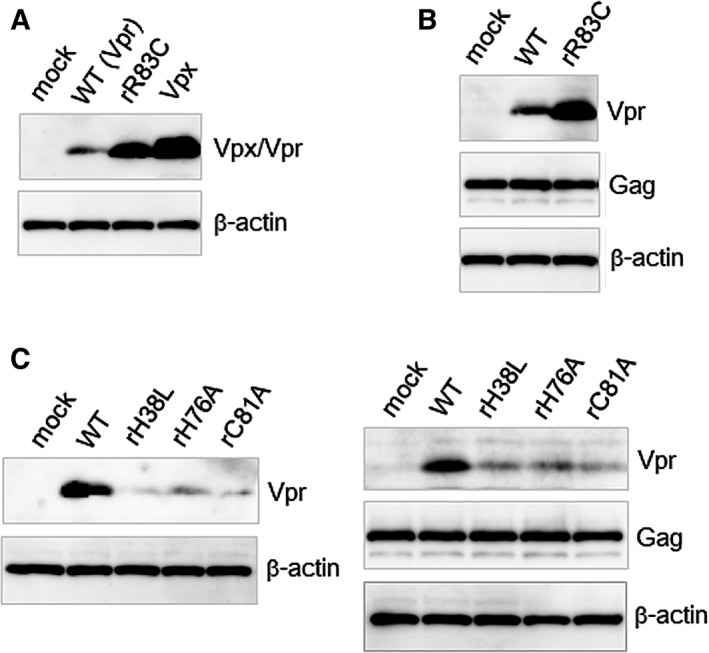
Expression levels of Vpr mutants carrying mutations in amino acids HHCR in HIV‐2 Vpr. (A) Immunoblot analysis of proteins in 293T cells transfected with an expression vector for HIV‐2 Vpr with a FLAG tag at its N terminus (pEF‐Fvpr2) 19, its mutant, or that for HIV‐2 Vpx with a FLAG tag at its N terminus (pEF‐Fvpx) 19 (25 ng). (B) Immunoblot analysis of proteins in 293T cells transfected with pEF‐Fvpr2 or its mutant (25 ng) with an expression vector for HIV‐2 Gag with a FLAG tag at its N terminus (pEF‐Fgag2) 22 (475 ng). (C) Immunoblot analysis of proteins in 293T cells transfected with pEF‐Fvpr2 or its mutant (25 ng) with the empty vector pEF1/*Myc*‐HisA or pEF‐Fgag2 (475 ng). In the transfections shown in (A, C left), pEF1/*Myc*‐HisA (475 ng) was added in addition to pEF‐Fvpx/pEF‐Fvpr2 or its mutants (25 ng). In the blotting shown in (A–C), anti‐FLAG M2 was used to detect Vpx, Vpr, and Gag, and anti‐β‐actin clone AC‐15 was used to detect β‐actin. The pEF1/*Myc*‐HisA was used as the mock sample.

We finally focused on the zinc‐binding sites in Vpx and Vpr. In Vpx, this site was first revealed by an X‐ray analysis 33, and it functions to prevent instability 22. The amino acids H39, H82, C87 and C89 in Vpx bind to zinc, forming an H2C2‐type zinc site. The corresponding amino acids in Vpr (GH‐123/GL‐AN strain) are H38, H76, C81 and R83 (Fig. 1D); notably, C81 is the only cysteine. This suggests that if, like Vpx, Vpr had a putative H2C2‐type zinc site, its expression level would increase. Therefore, we constructed the Vpr R83C mutant, in which R83 was changed to C, and examined its expression level. As shown in Fig. 2A, the amount of Vpr protein in the Vpr R83C mutant was higher than that in the WT. The amount of Vpr R83C was still a little smaller than the amount of Vpx, but this was likely caused in the stages prior to translation, as shown in Fig. 1B. An increase in the expression level of Vpr R83C was also observed in the presence of Gag (Fig. 2B). Next, H38, H76 and C81 of Vpr were changed to L, A and A, respectively, as in the previously reported mutants of Vpx, and the expression of each of the resultant mutants, H38L, H76A and C81A, was examined. As shown in Fig. 2C, the expression levels in these mutants were lower than that of WT Vpr with or without Gag. This result suggests that H38, H76 and C81 are involved with Vpr expression; however, the involvement of zinc in forming sites other than H2C2‐type sites remains unknown.

**Figure 3:**
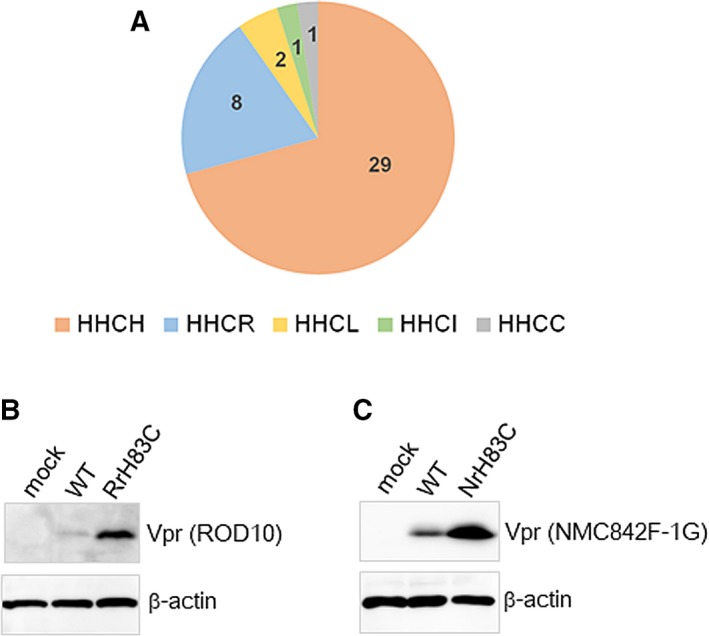
Expression levels of Vpr mutants carrying mutation HHCC in the amino acids HHCH in HIV‐2 Vpr. (A) Pie chart of various strains of HIV‐2 Vpr containing amino acids corresponding to the HHCC of Vpx. This contains all HIV‐2 Vpr sequences in Los Alamos HIV Sequence Database, 2016 (https://www.hiv.lanl.gov/content/sequence/HIV/mainpage.html). (B) Immunoblot analysis of proteins in 293T cells transfected with pEF‐FRvpr2 (expression vector for ROD10 Vpr) or its H83C mutant (25 ng). (C) Immunoblot analysis of proteins in 293T cells transfected with pEF‐FNvpr2 (expression vector for NMC842F‐1G Vpr with a FLAG tag at its N terminus) or its H83C mutant (25 ng). In the transfections shown in (B, C), pEF1/*Myc*‐HisA (475 ng) was added in addition to pEF‐FRvpr2/ pEF‐FNvpr2 or its mutants (25 ng). In the blotting shown in (B, C), anti‐FLAG M2 was used to detect Vpr and anti‐β‐actin clone AC‐15 was used to detect β‐actin. The pEF1/*Myc*‐HisA was used as the mock sample.

In our previous studies and this study, we have used GH‐123/GL‐AN as an HIV‐2 strain. Next, amino acids of Vpr corresponding to the HHCC of Vpx in the other strains were checked in the Los Alamos HIV Sequence Database, 2016, and summarized in Fig. 3A. As shown, the first three, HHC, are conserved in all strains. The last amino acid varies, but in 40 strains, it is not a cysteine. Exceptionally, one strain has cysteine, forming an HHCC motif like Vpx. HHCR, such as GH‐123, is the second biggest population. The biggest one is HHCH, including ROD10 23, NMC842F‐1G 24, and so on. Thus, we changed the fourth histidine (H83) in Vpr of these two strains to cysteine, making their mutants H83C, and examined their expression level. As shown in Fig. 3B (ROD10) and Fig. 3C (NMC842F‐1G), expression level of H83C is much higher than that of WT. The results show the significance of HHCR or HHCH residues in regulating the expression levels of Vpr/Vpx and that the high expression of Vpr is avoided by carrying the HHCC motif.

### Characterization of HIV‐2 having HHCC mutant Vpr

**Figure 4:**
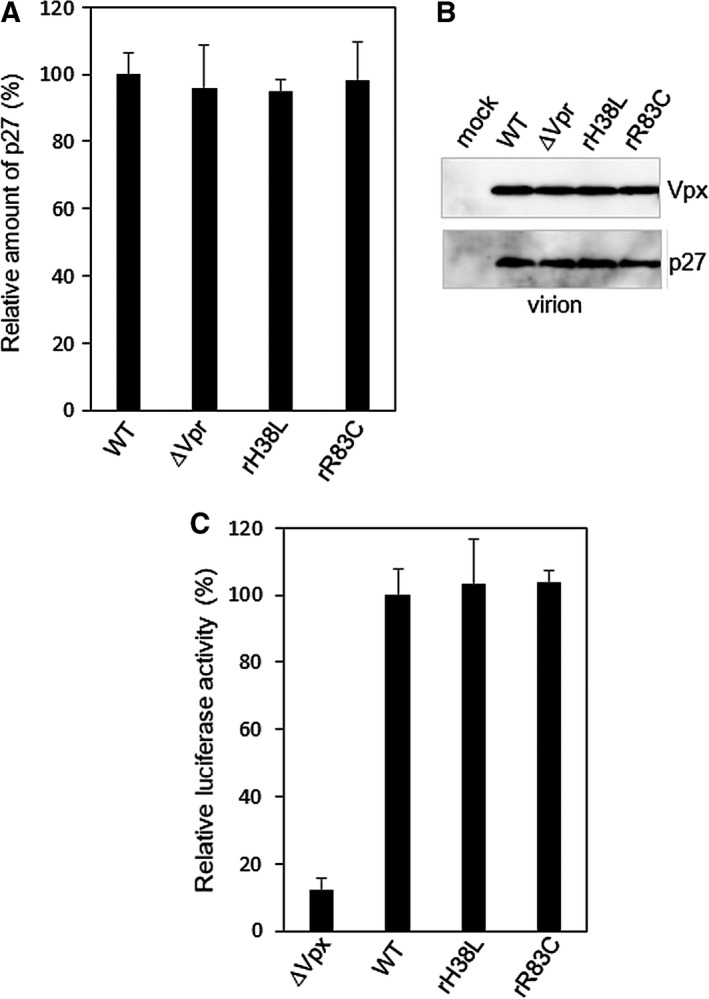
Characteristics of HIV‐2 with mutations in H38 and R83 of Vpr. (A) Amount of virus released from 293T cells transfected with HIV‐2 infectious clone pGL‐AN 21 or its *vpr* mutants. (B) Immunoblot analysis of proteins in the virions produced from 293T cells transfected with pGL‐AN or its *vpr* mutants. For detection, HIV‐2 Vpx mAb 6D2.6 and antiserum to SIV‐p27 were used. The empty vector pEF1/*Myc*‐HisA was used as the mock sample. (C) Relative luciferase activity of PMA‐differentiated THP‐1 cells infected with pseudo‐typed virus obtained from 293T cells transfected with pCMV‐G 27 and an *env*‐deficient HIV‐2 infections clone containing a *luc* gene, based on pGL‐AN, pGL‐Ns/luc 26, its *vpx*‐minus type 26 or its *vpr* mutants.

The R83C mutation was then introduced into the *vpr* gene of HIV‐2 infectious clone pGL‐AN 21, constructing pGL‐rR83C, and this vector was transfected into 293T cells. As controls, pGL‐Ec, which does not express Vpr 25, and the newly constructed pGL‐rH38L, which expresses Vpr H38L mutant with a low expression level (Fig. 2C), were used in addition to WT. The amounts of released virions in the supernatants were examined by p27 antigen ELISA. As shown in Fig. 4A, no clear difference was seen in the viral late phase. The released virions were prepared, and the Vpx protein incorporated in virions was analyzed by western immunoblotting, since Vpx and Vpr are known to be incorporated using the same Gag p6 motif 31. However, Vpx amounts were shown not to be affected by Vpr mutations (Fig. 4B). Finally, these point mutations were introduced into an *env*‐deficient luciferase reporter GL‐AN clone (pGL‐Ns/luc) 26, and these vectors were cotransfected into 293T cells with pCMV‐G, which expresses VSV‐G 27, by using the calcium phosphate coprecipitation method. Released pseudo‐typed viruses were normalized, and they were used to infect PMA‐differentiated THP‐1 22. Cells were lysed, and luciferase assays were performed. As shown in Fig. 4C, the virus infectivity of the Vpr mutant was almost the same as that of the WT. We have not obtained the difference of phenotype between WT and R83C yet, but further study using the Vpr R83C mutant that is aimed at revealing the virological significance of low level Vpr expression is in progress in our lab.

## Discussion

Here, we showed that the introduction of a putative H2C2‐type zinc‐binding site into Vpr increases its expression level. Our previous study revealed that the H2C2‐type zinc‐binding site of Vpx prevents instability of the protein. The presence of an H2C2 site in Vpx and the absence of this site in Vpr are highly conserved in HIV‐2. Among 41 strains in Los Alamos HIV Sequence Database, 2016, 40 have a C2H2‐type zinc‐binding site in Vpx. In contrast to that, its corresponding motif in Vpr is not C2H2 in 40 strains (Fig. 3A), but HHCH, HHCR, HHCL or HHCI. Worthy of note, one strain 34 exceptionally has H2C2‐type Vpr, although its virological significance is unclear. It seems that the large difference in expression level between Vpr and Vpx by HIV‐2 is determined, at least partially, by the absence or presence of this site. Vpx is known to degrade the anti‐viral host protein SAMHD1 13, 14 as well as to behave somehow SAMHD1‐independently 35, 36, which becomes important for virus replication in macrophages and T cells. A large amount of protein is likely required for Vpx to perform these functions. The presence of an H2C2‐type zinc‐binding site in Vpx to prevent protein degradation may help keep the protein level sufficiently high for this role. However, excessive overexpression of Vpx results in this protein losing its ability to degrade SAMHD1 32, which is similar to the observed phenomenon of HIV‐1 Vpr 37. Although Vpr is important for efficient virus replication in T cells, especially in the absence of Vpx 25, 38, knowledge of the functions of HIV‐2 Vpr remains incomplete. The low expression level of Vpr is thought to be sufficiently high to exert its functions and/or sufficiently low to prevent its detrimental effect on cells, but the details are under investigation.

All Vpx/Vpr proteins of SIVs/HIVs are thought to have simple structures consisting of three major helices; thus, they all have elements of intrinsic structural disorder. This leads to an advantage in functional revolution. However, the appropriate protein amount must be adjusted for each protein based on their specific functions and characteristics. Viruses may use zinc in a very simple way to regulate the expression level of Vpx/Vpr. Further work is needed to better understand the relationship between evolution and structural characteristics, such as zinc binding.

## Author contributions

RK, main experiments; MY and HIC, experiments; MO, data elucidation; MF, direction of the research and writing the manuscript.
